# Ketogenic diet and Parkinson’s disease

**DOI:** 10.3389/fnmol.2026.1914756

**Published:** 2026-08-05

**Authors:** Yuzhao Ye, Shuzhu Li, Tao Chen

**Affiliations:** Department of Neurology, Hainan General Hospital, Hainan Affiliated Hospital of Hainan Medical University, Haikou, China

**Keywords:** degenerative disease, ketogenic diet, ketones, Parkinson’s disease, β-hydroxybutyrate

## Abstract

Parkinson’s disease (PD), a common degenerative disorder in middle-aged and elderly individuals, has an unclear etiology and pathogenesis, and there is currently no cure. In recent years, the ketogenic diet (KD), a dietary regimen characterized by high-fat, extremely low carbohydrates, and moderate protein intake, has shown promise in the treatment of PD. This review systematically analyzes the multiple mechanisms through which KD exerts its effects in PD, such as energy metabolism reprogramming, neuroinflammation suppression, epigenetic regulation, and modulation of the brain-gut axis. The aim of this review is to provide theoretical foundations and practical guidance for the clinical application of the ketogenic diet as an adjunctive therapeutic strategy for PD.

## 1 Introduction

Parkinson’s disease (PD) is a common neurodegenerative disorder predominantly affecting older adults (Tolosa et al., 2021). Its clinical manifestations include motor symptoms such as bradykinesia, resting tremor, and rigidity, as well as a range of non-motor symptoms including hyposmia, constipation, and sleep disturbances (Morris et al., 2024). The pathogenesis of PD is highly complex and involves multiple factors, including genetic susceptibility, environmental exposures, mitochondrial dysfunction, neuroinflammation, oxidative stress, and impaired proteostasis. Increasing evidence also indicates that gut microbiota dysbiosis may contribute to the onset and progression of PD (Breen et al., 2019). Current therapeutic strategies for PD primarily rely on dopamine replacement therapies and deep brain stimulation (Elsworth, 2020), which can alleviate symptoms but do not prevent disease progression. Thus, identifying interventions capable of modifying the underlying disease process has become a critical focus of PD research. The ketogenic diet (KD), a high-fat, low-carbohydrate dietary regimen with adequate protein intake, was originally developed in the 1920’s as a metabolic therapy for refractory epilepsy. Owing to its well-established efficacy in controlling seizures, KD has remained an established treatment for drug-resistant epilepsy for more than a century. Its potential therapeutic mechanisms and clinical benefits in PD have also garnered substantial attention. This review provides a comprehensive summary of recent advances in the application of the ketogenic diet in PD, with particular emphasis on its neuroprotective mechanisms and clinical efficacy, aiming to provide a scientific rationale for its future translation into clinical practice.

## 2 Ketogenic diet

**FIGURE 1:**
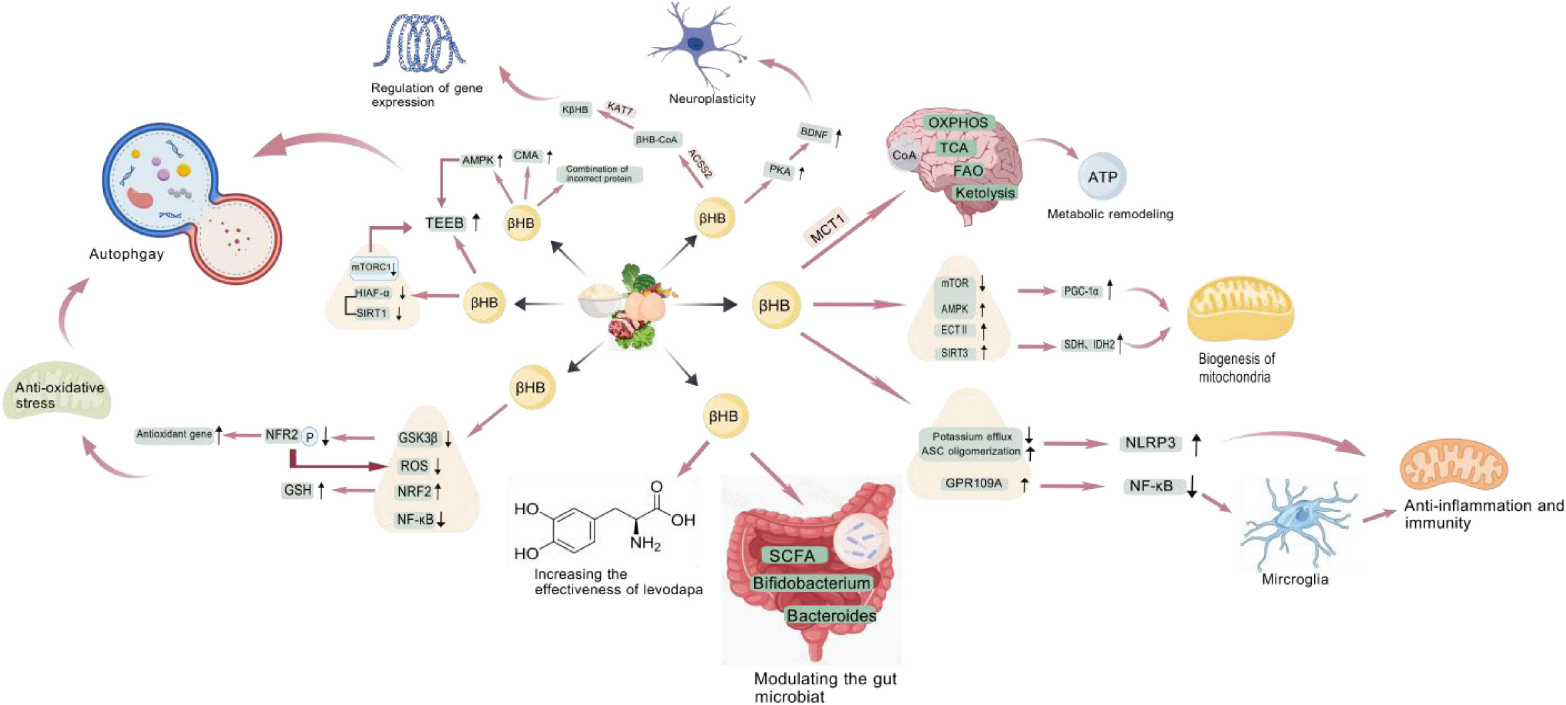
Integrated mechanisms of ketogenic diet specifically influences Parkinson’s disease through regulating aspects such as oxidative stress, mitophagy, and neuroinflammation (Jiang et al., 2025).

The ketogenic diet (KD) is a dietary regimen characterized by a significantly reduced carbohydrate intake and increased consumption of fats and proteins (Mazandarani et al., 2023). Through the modification of the macronutrient composition, KD stimulates the generation of ketone bodies, thus emulating the metabolic state observed during fasting. In the conventional KD protocol, fats account for approximately 70%–85% of the total caloric intake, carbohydrates are limited to 3%–5%, and proteins make up around 10%–20% (Roehl and Sewak, 2017), with a macronutrient ratio of fats to the combined proportion of proteins and carbohydrates being 4:1. From a physiological perspective, under this dietary pattern, severe carbohydrate restriction restricts glucose availability, compelling the body to shift its primary metabolic fuel from glucose to fatty acids. Subsequently, these fatty acids are metabolized into ketone bodies, including acetoacetate, acetone, and β-hydroxybutyrate, thereby increasing ketogenesis (Knight et al., 2022). The body gradually enters a state of “nutritional ketosis” (Monda et al., 2024), which is fundamentally distinct from pathological ketoacidosis. The levels of ketone bodies produced during KD intervention do not disrupt the systemic acid-base balance and represent a physiologically regulated metabolic state (Li et al., 2025). Due to their distinctive biochemical characteristics, ketone bodies can easily cross the blood-brain barrier, offering an alternative energy source to maintain neuronal metabolic homeostasis. Accumulating evidence suggests that the ketogenic diet (KD) exerts diverse effects in Parkinson’s disease (PD), encompassing the remodeling of energy metabolism, the enhancement of mitochondrial function, the suppression of neuroinflammation, the alleviation of oxidative stress, the promotion of autophagy and proteostasis, the modulation of the gut microbiota, the regulation of epigenetic processes and neuronal plasticity, and the improvement of the therapeutic efficacy of levodopa. Via these mechanisms, KD may impact both the onset and progression of PD (Figure 1).

## 3 Mechanistic basis of the multi-target effects of the ketogenic diet in Parkinson’s disease

### 3.1 Metabolic reprogramming

Energy metabolism is essential for maintaining normal neuronal function. Under physiological conditions, glucose metabolism, which occurs through glycolysis and the tricarboxylic acid (TCA) cycle, generates adenosine triphosphate (ATP) to support neuronal activity. In patients diagnosed with Parkinson’s disease (PD), cerebral glucose metabolism is typically reduced, especially in the premotor cortex and parietal association areas (Huang et al., 2007). Research has indicated that the activities of key enzymes involved in glucose metabolism pathways are altered in the PD brain. For instance, the decreased activities of hexokinase (HK) and pyruvate kinase (PK) impede the conversion of glucose to pyruvate, resulting in reduced ATP production, accumulation of lactate and pyruvate, and ultimately insufficient energy supply (Ostrakhovitch et al., 2025). Additionally, the activities of citrate synthase and isocitrate dehydrogenase, two crucial enzymes in the TCA cycle, are significantly reduced, further diminishing metabolic efficiency.

β-hydroxybutyrate (β-HB), one of the principal ketone bodies, serves as an important alternative energy substrate for the brain in PD. It enters the brain through monocarboxylate transporter 1 (MCT1), expressed on microvascular endothelial cells and astrocytes. This transport process depends on circulating ketone body concentrations rather than neuronal activity. MCTs are proton-linked membrane carriers responsible for transporting monocarboxylates, including ketone bodies, and currently represent the primary transporters for ketone bodies, with widespread distribution throughout the brain (Jensen et al., 2020). Upon entering the brain, ketone bodies are converted to acetyl-CoA and subsequently fuel ATP production by enhancing oxidative phosphorylation, the TCA cycle, mitochondrial fatty acid β-oxidation, and ketolysis pathways (Miller et al., 2018). This metabolic shift not only increases the efficiency of ATP generation but also improves neuronal energy availability. Particularly under conditions of impaired glucose utilization, the capacity of ketone bodies to sustain neuronal energy metabolism becomes particularly important.

### 3.2 Optimization of mitochondrial function

In animal models, inhibition of mitochondrial complex I activity by neurotoxins induces Parkinsonian syndromes, providing strong evidence for the mechanistic link between mitochondrial dysfunction and Parkinson’s disease (PD). Reduced complex I activity has been reported not only in patients with PD but also in toxin-induced models and genetically engineered *in vitro* and *in vivo* PD models (González-Rodríguez et al., 2021).

Studies have shown that β-HB promotes mitochondrial biogenesis by inhibiting the mechanistic target of rapamycin (mTOR) signaling pathway and activating AMP-activated protein kinase (AMPK). Once activated, AMPK upregulates the expression of peroxisome proliferator–activated receptor γ coactivator-1α (PGC-1α) (Gano et al., 2014). PGC-1α interacts with nuclear receptors such as PPARs to activate the transcription of mitochondria-related genes, including those encoding respiratory chain complexes, transport proteins, and metabolic enzymes, ultimately promoting mitochondrial biogenesis and improving mitochondrial function in neurodegenerative diseases such as PD and Alzheimer’s disease. Additionally, after entering mitochondria, ketone bodies undergo metabolic processing that enhances electron flow through the electron transport chain, thereby enhancing its efficiency. Ketone bodies may also increase SIRT3 expression, which subsequently deacetylates and activates key metabolic enzymes, improves mitochondrial metabolic capacity, preserves mitochondrial membrane stability, and inhibits the opening of the mitochondrial permeability transition pore (Newman and Verdin, 2017), collectively contributing to the optimization of mitochondrial function.

### 3.3 Attenuation of oxidative stress

Oxidative stress represents another major consequence of impaired energy metabolism in Parkinson’s disease (PD). Damage to components of the mitochondrial respiratory chain, together with aberrant dopamine metabolism, leads to excessive production of reactive oxygen species (ROS), thereby overwhelming the endogenous antioxidant defense system. During dopamine auto-oxidation, the neuromelanin formed contains reduced iron ions, which participate in Fenton reactions with hydrogen peroxide generated during dopamine metabolism, producing highly cytotoxic hydroxyl radicals that trigger lipid peroxidation and neuronal apoptosis. Studies have demonstrated significantly elevated levels of carbonylated proteins within mitochondrial fractions of PD brains, providing direct evidence of oxidative injury (Keeney et al., 2006). Oxidative stress not only damages essential biomolecules—including lipids, proteins, and nucleic acids—but also exacerbates neuronal injury by activating inflammatory responses and apoptosis pathways. Moreover, overexpression of α-synuclein further amplifies oxidative stress, forming a vicious cycle that accelerates disease progression (Park et al., 2025). Experimental studies have shown that antioxidant interventions partially ameliorate pathological features in PD animal models (Tng et al., 2025), further underscoring the critical role of oxidative stress in PD pathogenesis. Thus, oxidative stress is not merely a downstream consequence of metabolic dysfunction but also an active driver of PD progression.

β-HB has been reported to reduce intracellular ROS (Haces et al., 2008), thereby mitigating oxidative damage. In addition, β-HB inhibits glycogen synthase kinase-3β (GSK-3β) activity, reducing phosphorylation-dependent degradation of nuclear factor erythroid 2–related factor 2 (NRF2), increasing its stability and facilitating its nuclear translocation to promote antioxidant gene expression. Glutathione (GSH), a tripeptide ubiquitously expressed in cells, plays a central role in maintaining redox homeostasis. Activation of the NRF2 pathway elevates intracellular GSH levels, a process dependent on the mild oxidative and electrophilic stress induced by KD, which triggers adaptive cytoprotective responses to restore redox balance (Milder et al., 2010). Although not derived from PD populations, magnetic resonance spectroscopy studies in patients with epilepsy have shown that KD therapy enhances de novo GSH synthesis in the brain and improves cerebral redox status, providing indirect evidence that KD may promote antioxidant capacity through metabolic adaptation (Napolitano et al., 2020). Similarly, in PD rat models, KD increased striatal GSH levels and protected dopaminergic neurons from 6-hydroxydopamine (6-OHDA)–induced neurotoxicity (Cheng et al., 2009). Together, these findings suggest that KD-induced redox modulation, including NRF2 activation and enhanced GSH synthesis, may contribute to neuroprotection in PD, although direct clinical evidence in PD remains limited.

Furthermore, β-HB suppresses the nuclear factor-κB (NF-κB) signaling pathway, reducing pro-inflammatory cytokine release and indirectly alleviating oxidative stress. As an efficient energy substrate, ketone bodies also improve mitochondrial function and reduce ROS generation (Gómora-García et al., 2023), while the activation of autophagy promotes the clearance of damaged mitochondria, collectively contributing to the attenuation of oxidative stress.

### 3.4 Neuroinflammation and immune modulation

Microglia, the resident immune cells of the central nervous system, play a pivotal role in PD-related neuroinflammation. Misfolded and aggregated α-synuclein can activate microglia, which initially exert a protective role by phagocytosing aberrant proteins (Shi and Yong, 2025). However, under conditions of sustained stimulation, microglia progressively shift toward a pro-inflammatory phenotype, releasing a variety of inflammatory mediators that exacerbate dopaminergic neuronal injury. Damaged neurons subsequently release damage-associated molecular patterns, further amplifying microglial activation and forming a vicious cycle of “microglial activation–exacerbated neuroinflammation–neuronal injury” (Xue et al., 2025). In addition, neuroinflammation in PD also involves activation of astrocytes (Kam et al., 2020). Extracellular α-synuclein can activate the nuclear factor-κB (NF-κB) and p38 mitogen-activated protein kinase (MAPK) signaling pathways in astrocytes, promoting the release of pro-inflammatory cytokines and reactive oxygen species, which further injure dopaminergic neurons (Chou et al., 2021; Lee et al., 2023). Postmortem analyses of PD brains have confirmed the presence of reactive astrocytes exhibiting neurotoxic, pro-inflammatory phenotypes (Liddelow et al., 2017). Although peripheral immune cell infiltration may contribute to the inflammatory milieu, its overall influence appears relatively limited.

In PD animal models, KD exerts anti-inflammatory effects by suppressing microglial activation and reducing the release of inflammatory cytokines (Zhu et al., 2022). Studies indicate that β-HB activates the anti-inflammatory G protein-coupled receptor 109A (GPR109A) pathway, promoting intracellular calcium influx and inhibiting endoplasmic reticulum stress. This subsequently suppresses NF-κB activation and the expression of other pro-inflammatory mediators, limiting excessive microglial activation and decreasing levels of pro-inflammatory cytokines such as tumor necrosis factor-α (TNF-α), interleukin-1β (IL-1β), and interferon-γ (IFN-γ), thereby mitigating neuroinflammation (Zhang et al., 2021). Furthermore, β-HB restricts potassium efflux and reduces oligomerization and speck formation of apoptosis-associated speck-like protein containing a caspase recruitment domain (ASC), thereby inhibiting the initiation and assembly of the nucleotide-binding oligomerization domain-like receptor protein 3 (NLRP3) inflammasome. This leads to decreased secretion of IL-1β and IL-18 (Youm et al., 2015). Collectively, these findings suggest that KD may exert neuroprotective effects by suppressing M1-type microglial activation and downregulating pro-inflammatory cytokine expression.

### 3.5 Proteostasis and autophagy

Proteostatic dysregulation in PD is primarily associated with aberrant aggregation of α-synuclein and dysfunction of protein degradation pathways. α-Synuclein is the central pathogenic protein in PD; in the brains of affected individuals, it undergoes misfolding and forms soluble oligomers and insoluble fibrillar aggregates (Rodger et al., 2023). These aggregates progressively accumulate to form Lewy bodies, ultimately leading to neuronal dysfunction and cell death. Intracellular protein degradation systems—such as the ubiquitin–proteasome system and the autophagy–lysosomal pathway—are responsible for eliminating misfolded or abnormal proteins. In PD, impaired degradation capacity results in the ineffective clearance of α-synuclein and other aberrant proteins, causing their intracellular accumulation and disrupting proteostasis (Morrone Parfitt et al., 2024). In addition, gene duplications or mutations, mitochondrial dysfunction, and exacerbated oxidative stress further promote pathological α-synuclein aggregation and proteostatic imbalance.

Ketogenic diet helps maintain intracellular proteostasis through attenuation of oxidative stress and enhancement of autophagy. Regarding oxidative stress regulation, KD improves mitochondrial function, reduces oxidative protein damage, and preserves intracellular redox homeostasis and energy supply, thereby providing a favorable environment for proper protein folding by molecular chaperones. Autophagy, a lysosome-mediated cellular process, degrades endogenous components (including misfolded or aggregated proteins and damaged organelles) and exogenous pathogens (such as bacteria, viruses, and parasites). By preventing the accumulation of misfolded proteins, autophagy indirectly improves the protein-folding environment and maintains intracellular proteostasis. Studies have shown that β-HB inhibits mTORC1 signaling and upregulates SIRT1 expression, thereby promoting autophagy and regulating inflammatory responses in the brain (Chu et al., 2024; Gómora-García et al., 2023). Additionally, β-HB activates AMP-activated protein kinase (AMPK), directly triggering autophagy-related cascades and promoting the degradation of intracellular misfolded proteins (Puchalska and Crawford, 2017). β-HB also facilitates the clearance of oxidatively damaged proteins by promoting chaperone-mediated autophagy (CMA) (Bourdenx et al., 2021). Transcription factor EB (TFEB), a master regulator of lysosomal biogenesis, is upregulated by KD, thereby enhancing autophagic activity (Jiang et al., 2022). Multiple studies have demonstrated that mTORC1, in its basal active state, negatively regulates metabolic processes by directly phosphorylating and inhibiting TFEB (at Ser211 and Ser142) and TFE3 (at Ser321) (Roczniak-Ferguson et al., 2012). Further evidence indicates that the transcriptional activities of TFEB and TFE3 are mTORC1-independent but AMPK-dependent, whereas their nuclear translocation is solely regulated by mTORC1 (Paquette et al., 2021). Under starvation conditions, lysosomal calcium release increases cytosolic Ca^2 +^ levels, activating calcineurin (CaN), which dephosphorylates TFEB at Ser142 and Ser211 (Franco-Juárez et al., 2022), promoting its nuclear translocation and subsequent activation of the autophagy–lysosomal system. Moreover, β-HB can directly bind to misfolded proteins, enhancing their solubility and structural stability, thereby facilitating their degradation or assisting in their refolding into correct conformations (Madhavan et al., 2025).

### 3.6 Brain–gut axis interactions

The brain–gut axis refers to the bidirectional communication network between the central nervous system and the gastrointestinal tract mediated by neural, endocrine, immune, and microbial signaling pathways (Cryan et al., 2019), in which the gut microbiota serves as a critical regulatory hub. A growing body of evidence suggests that bidirectional communication along the brain–gut axis is involved in PD pathogenesis (McFleder et al., 2023). Indeed, many patients exhibit abnormal α-synuclein aggregation within the enteric nervous system (ENS). Based on the distribution pattern of α-synuclein deposition, Braak et al. (2003) proposed the hypothesis that PD may originate in the gastrointestinal tract and spread to the brain in a prion-like manner via the vagus nerve. Studies in PD patients and animal models have demonstrated neuronal and glial injury within the ENS. Sampson et al. (2016) further confirmed that transplantation of gut microbiota from PD patients into genetically susceptible mice exacerbated motor deficits in the recipient animals . Alterations in the gut microbiota are associated with an increased risk of developing PD, among which reduced abundance and diversity of short-chain fatty acid (SCFA)-producing bacteria—particularly propionate- and butyrate-producing species—represent a characteristic microbial signature of PD (Baert et al., 2021).

Ketogenic diet modulates the brain–gut axis by altering gut microbial composition and diversity. β-HB has been reported to inhibit the growth of Bifidobacterium. *In vitro* studies demonstrate that β-HB suppresses *Bifidobacterium* proliferation in a dose-dependent manner, an effect primarily mediated through reductions in culture medium pH (Ang et al., 2020). Although these observations were obtained in non-PD settings, studies in both humans and mice have shown that KD or exogenous ketone ester supplementation decreases the abundance of *Bifidobacterium* while increasing the abundance of *Bacteroides*, suggesting a conserved microbial response to ketosis across different physiological and disease contexts (Swidsinski et al., 2017). Similarly, studies in obesity models have demonstrated that KD reshapes gut microbial composition, characterized by reduced abundances of Firmicutes and Actinobacteria and an increased abundance of Proteobacteria (Li X. et al., 2024). Some studies have also observed a decline in microbial diversity following KD, indicating reduced richness of microbial taxa. The decrease in *Bifidobacterium* is associated with reduced proportions of intestinal Th17 cells, which are pro-inflammatory immune cells, suggesting that KD-induced microbial shifts may ameliorate inflammatory responses (Ang et al., 2020). This mechanism supports the notion that ketone bodies indirectly modulate host immune function by altering gut microbial communities (David et al., 2014). Although these findings are largely derived from non-PD studies, they provide mechanistic insight into how KD-mediated remodeling of the gut microbiota may influence brain–gut communication and potentially contribute to neuroprotection in PD. A clinical study further reported that KD increases both SCFA-producing bacteria and the substrates that support SCFA production (Beam et al., 2021). SCFAs possess anti-inflammatory and metabolic regulatory properties and play an important role in modulating host immunity. Collectively, these findings indicate that ketone bodies can influence intestinal microecological balance and host immune function by inhibiting specific microbial taxa, altering microbial composition, and modifying microbial diversity.

### 3.7 Epigenetic regulation and neural plasticity

DNA methylation represents a major epigenetic regulatory mechanism that modulates gene expression by adding methyl groups to CpG islands (Tang et al., 2022). In PD, hypomethylation of the *SNCA* promoter region results in the overexpression of α-synuclein, thereby facilitating its pathological aggregation and accelerating neuronal injury (Schmitt et al., 2024). Aberrant methylation of iron-metabolism–related genes such as *SLC7A11* can impair cystine uptake and glutathione synthesis, increasing susceptibility to ferroptosis (Gao et al., 2025) and further exacerbating neurodegeneration. Histone modifications (e.g., acetylation and methylation) also participate in PD pathophysiology by altering chromatin structure and gene accessibility. Histone deacetylase (HDAC) inhibitors have been shown to ameliorate both motor and non-motor manifestations in PD animal models by maintaining a relaxed chromatin state, thereby activating neuroprotective gene transcription and reducing dopaminergic neuronal degeneration (Li H. et al., 2024). Environmental factors such as pesticide exposure may induce changes in DNA methylation and histone modification, suppressing the expression of neuroprotective genes and consequently increasing disease susceptibility. Conversely, smoking and caffeine intake may exert neuroprotective effects through epigenetic pathways. Multiple studies have demonstrated the unique epigenetic actions of β-HB (Newman and Verdin, 2014). As an endogenous HDAC inhibitor (Gregoretti et al., 2004), β-HB modulates various histone post-translational modifications, including histone methylation and acetylation. For example, β-HB can induce hyperacetylation of histones at the promoter region of forkhead box O3 (Foxo3a), enhancing its transcriptional expression (Shimazu et al., 2013). Foxo3a is the mammalian homolog of DAF-16, a stress-responsive transcription factor regulating lifespan in *Caenorhabditis elegans*. Activation of the DAF-16/FOXO signaling cascade promotes the transcription of neuroprotective genes. Experimental evidence further indicates that β-HB administration can ameliorate the phenotype of chemically induced PD in mice (Tieu et al., 2003). As the principal ketone body, β-HB can be converted into β-hydroxybutyryl-CoA (β-HB-CoA) via acyl-CoA synthetase short-chain family member 2 (ACSS2). This intermediate can subsequently be utilized by lysine acetyltransferase 7 (KAT7) and related enzymes to catalyze histone β-hydroxybutyrylation (Kbhb)—predominantly occurring at K9 and K27 residues of histone H3 (Wang et al., 2025)—thereby modulating chromatin architecture and gene expression. In addition, studies have revealed that KD promotes neural plasticity by modulating the cortical synaptic proteome, particularly through activation of the PKA signaling pathway, which enhances the expression of brain-derived neurotrophic factor (BDNF) (Acuña-Catalán et al., 2024). β-HB-mediated regulation of histone modifications may further potentiate BDNF transcription. As a key neurotrophic factor, BDNF supports neuronal differentiation, synaptogenesis, and inhibition of apoptosis, ultimately exerting robust neuroprotective effects (Colucci-D’Amato et al., 2020). A substantial body of evidence demonstrates that β-HB significantly elevates BDNF levels both *in vivo* and *in vitro*. A research team from Xi’an Jiaotong University reported that β-HB supplementation in mice fed a normal diet markedly increased BDNF concentrations in serum and hippocampal tissue, accompanied by improvements in learning and memory performance (Hu et al., 2020).

### 3.8 Enhancement of levodopa bioavailability

The principal pathological hallmark of Parkinson’s disease is the progressive degeneration of dopaminergic neurons within the nigrostriatal pathway. As a biochemical precursor of dopamine, levodopa can readily cross the blood–brain barrier (BBB) and is subsequently decarboxylated by aromatic L-amino acid decarboxylase to dopamine, thereby directly compensating for impaired dopaminergic neurotransmission.

In clinical practice, levodopa absorption in the intestine competes with neutral amino acids for shared transporters. KD, by restricting carbohydrate intake and limiting the availability of competing large neutral amino acids (LNAAs), may enhance intestinal levodopa absorption and improve its bioavailability (Rusch et al., 2023), thus enhancing levodopa absorption and improving its bioavailability. Moreover, ketone bodies derived from KD serve as an alternative energy substrate for the brain, optimizing neuronal energy metabolism. A more favorable metabolic milieu may improve the cerebral utilization of levodopa (Pokora et al., 2025), indirectly improving central drug utilization efficiency and potentially alleviating long-term treatment-related complications such as motor fluctuations and dyskinesias.

## 4 Preclinical and clinical evidence supporting the ketogenic diet

As a high-fat, low-carbohydrate dietary regimen, KD has accumulated multifaceted evidence supporting its neuroprotective potential in PD (Table 1). Mechanistically, the medium-chain triglyceride ketogenic diet (MCT-KD) has been shown to markedly attenuate dopaminergic neuronal injury in 1-methyl-4-phenyl-1,2,3,6-tetrahydropyridine (MPTP)-induced PD mouse models. It exerts antioxidative effects through activation of the PI3K/AKT/NRF2 signaling pathway, reduces mitochondrial damage, and suppresses microglial activation, thereby preserving dopaminergic neuronal integrity (Zhang et al., 2023). In addition, MCT-KD has been reported to modulate the gut microbiota and its metabolites, restore purine metabolic homeostasis within the substantia nigra, and subsequently ameliorate PD-related phenotypes. At the clinical level, a randomized controlled trial involving 78 participants with PD demonstrated that adjunctive KD intervention for six months, in combination with standard pharmacotherapy, resulted in significantly greater improvements in Unified Parkinson’s Disease Rating Scale (UPDRS) scores, activities of daily living, and motor performance compared with the control group (Juan, 2018). A recent exploratory 12-week study further confirmed that a modified low-dairy KD effectively sustained nutritional ketosis and improved UPDRS-III scores, dynamic balance, and quality-of-life measures, without exacerbating constipation or causing excessive weight loss (Worster et al., 2025). Another preliminary randomized controlled trial similarly indicated that an 8-week KD regimen is safe and, notably, more effective in improving non-motor symptoms (Phillips et al., 2018). Collectively, current preclinical and clinical findings suggest that KD may represent a feasible adjunctive therapeutic strategy for PD, with potential benefits in alleviating both motor and non-motor symptoms and potentially modifying disease progression. Nevertheless, limitations remain, including small sample sizes and relatively short follow-up durations. Large-scale, long-term studies are needed to clarify its sustained efficacy, optimal implementation protocols, and underlying molecular mechanisms.

**TABLE 1:** Summary of clinical studies on ketogenic diet (KD) in Parkinson’s disease (PD).

References	Study design	Participants	Intervention	Duration	Primary outcomes	Main findings	Limitations
Juan, 2018	Randomized controlled trial	78 patients with PD (39 KD vs. 39 control)	Ketogenic diet plus standard pharmacotherapy vs. standard pharmacotherapy alone	6 months	UPDRS, activities of daily living, motor function	Adjunctive KD significantly unproved UPDRS scores, daily living activities, and motor performance compared with the control group.	Single-center study; relatively small sample size; published in Chinese; limited mechanistic evaluation.
Phillips et al., 2018	Pilot randomized controlled trial	Patients with PD	Ketogenic diet vs. low-fat diet	8 weeks	Motor and non-motor symptoms, safety	Both diets were safe, while KD produced greater improvements in non-motor symptoms, particularly urinary problems, pain, fatigue, daytime sleepmess, and cognitive impairment.	Small sample size; short intervention duration; pilot study.
Worster et al., 2025	Mixed-methods feasibility study	Patients with PD	Modified low-dairy ketogenic diet	12 weeks	Feasibility, nutritional ketosis. UPDRS-III. balance, quality of life	Improvements were observed in UPDRS-III. dynamic balance, and quality of life without worsening constipation or excessive weight loss.	Exploratory design; no large control group; short follow-up.

## 5 Challenges and future perspectives

Although KD has demonstrated therapeutic potential in PD, several critical challenges hinder its clinical translation. Most existing clinical studies are limited by small sample sizes, short follow-up periods, and insufficiently rigorous control designs, underscoring the urgent need for large-scale, long-term randomized controlled trials. Additionally, the long-term safety of KD warrants further evaluation. Previous studies have indicated potential adverse metabolic effects—including dyslipidemia, nephrolithiasis, and osteoporosis—and the risk–benefit profile of KD in individuals with PD remains to be fully elucidated.

Future research should focus on the following directions: (1) conducting multicenter randomized controlled trials to systematically assess the effects of KD on PD symptoms and disease progression; (2) utilizing bioinformatics approaches and experimental models to further elucidate its neuroprotective mechanisms; (3) integrating genomic, metabolomic, and gut microbiome data to develop predictive biomarkers that support personalized nutritional interventions; and (4) exploring synergistic benefits between KD and non-pharmacological therapies, such as exercise interventions. Notably, considerable interindividual variability may exist in the responses of patients with PD to KD. Such heterogeneity may arise from differences in genetic background, gut microbiota composition, and metabolic phenotypes. Identifying patients most likely to benefit is therefore essential for implementing precision nutrition strategies (Balck et al., 2025; Qin et al., 2025).

Despite these challenges, KD is emerging as a valuable non-pharmacological intervention within the comprehensive management of PD. With continued mechanistic investigation and refinement of individualized approaches, KD holds promise as an adjunct to existing therapeutic modalities, offering new avenues for optimizing disease management and improving quality of life.

## 6 Conclusion

As a metabolic intervention strategy, the ketogenic diet exhibits multifaceted therapeutic potential in the management of PD. By improving cerebral energy metabolism, suppressing neuroinflammation, and modulating the gut–brain axis, it may not only alleviate both motor and non-motor symptoms of PD but also may potentially exert disease-modifying effects. However, the long-term efficacy and safety of KD in PD patients remain to be validated in larger-scale clinical trials. During implementation, individualized dietary planning, multidisciplinary management, and regular monitoring are essential to ensure its safe and effective implementation. Future research should focus on conducting high-quality clinical trials, elucidating underlying mechanisms, and developing biomarker-based personalized strategies to facilitate the clinical translation of ketogenic interventions in PD. In summary, the ketogenic diet provides a novel adjunctive therapeutic approach beyond traditional pharmacological treatments for PD. With advancements in precision medicine and nutritional neuroscience, its role in the comprehensive management of PD is expected to become increasingly significant.
